# Chemicals and microbes in bioaerosols from reaction tanks of six wastewater treatment plants: survival factors, generation sources, and mechanisms

**DOI:** 10.1038/s41598-018-27652-2

**Published:** 2018-06-19

**Authors:** Yanjie Wang, Huachun Lan, Lin Li, Kaixiong Yang, Jiuhui Qu, Junxin Liu

**Affiliations:** 1 State Key Laboratory of Environmental Aquatic Chemistry, Research Center for Eco-Environmental Sciences, Chinese Academy of Sciences, Beijing, 100085 China; 2 National Engineering Laboratory for VOCs Pollution Control Material & Technology, University of Chinese Academy of Sciences, Beijing, 101408 P. R. China; 3 School of Environment, Tsinghua University, Beijing, 100084 China

## Abstract

Sampling was conducted from biochemical reaction tanks of six municipal wastewater treatment plants in the Yangtze River and Zhujiang deltas and the Jing-Jin-Ji region to assess their morphology, level, and composition. Morphological observations suggested that particles were scattered amorphously with C, O, and Si as the major elements. Bioaerosols are composed of spatially varying levels of microorganisms and chemicals. As the sampling height increased, the level of the components in the bioaerosols decreased. Wastewater in the biochemical reaction tanks was identified as an important source of bioaerosols using SourceTracker analysis. The aerosolization of film drops produced by bursting of bubbles was the main reason for the generation of bioaerosols. Increasing the aeration rate of water may promote bioaerosol generation. Relative humidity, temperature, wind speed, and solar illumination influenced the survival of bioaerosols. Large particle sedimentation and wind diffusion significantly decreased the atmospheric aerosol concentration. When the sampling point height increased from 0.1 m to 3.0 m, the concentrations of the microorganisms and total suspended particles decreased by 23.71% and 38.74%, respectively. Considerable attention should be paid to the control of total suspended particles and microorganisms in bioaerosols.

## Introduction

The aerated basin in a municipal wastewater treatment plant (WWTP) is a treatment pond in which microbial action is used to remove dissolved and suspended organic compounds in wastewater. Artificial aeration is often provided to promote the biological process. Injection of compressed air via submerged diffusers is among the most popular methods of aerating basins. Fine or medium bubble diffusers are utilized to provide oxygen and mix the process water. Raw wastewater contains a large diversity of microorganisms, such as bacteria, archaea, fungi, viruses, and protozoans. These microorganisms may be aerosolized during aeration processes. WWTPs have been identified as one of the sources for bioaerosol emission^1–3^. As metabolites, toxins, and fragments of microorganisms, including pathogenic microorganisms, may exist in bioaerosols, their emission is closely correlated with air pollution and health hazards for on-site workers and nearby residents^4–6^. International interest in bioaerosols from WWTPs has increased rapidly resulting in a broadening of the knowledge available regarding their identification, quantification, distribution, and health impacts. In the study of bioaerosol characteristics generated by WWTPs, 10^1^ to 10^4^ CFU/m^3^ microbes in bioaerosols were found by Gotkowska-Płachta’s research group^7^. Similar studies have shown that up to 1697 CFU/m^3^ and 930 CFU/m^3^ of culturable bacteria and fungi, respectively, exist in bioaerosols in a WWTP that used the A^2^/O treatment process^8^. High concentrations (up to 10^5^–10^7^ CFU/m^3^) have been measured in polluted areas, e.g., WWTPs^3^. Bioaerosols might also contain Gram-negative bacteria, such as *Giardialamblia*, *Cryptosporidium sp*., *Salmonella sp*., and *Shigella sp*., which could release endotoxins and affect the health of employees, particularly those working at WWTPs and community around^5^.

In the same range of bioaerosol microbial concentration, the exposure hazard quotient of bioaerosols ranged from 10^−3^ to 10^−2^ in a one-day exposure risk assessment^9^. Although the present model provides acceptable low risk values, it is worth noting that the risk will increase with the accumulation of pathogenic bacteria. Previous estimates have also suggested that exposure to airborne pathogens from wastewater can potentially place people at serious risk of cardiovascular and respiratory disease^10^. Particles, particularly those of small size, are easily spread to the atmosphere over distances and can cause infection in on-site workers as well as downwind residents.

Unlike those released from the soil surface, bioaerosols generated from water sources are usually surrounded by a thin layer of moisture. In addition to microbes, particles and chemicals such as ions and organics are also present in bioaerosols. Reports on bioaerosols emitted from WWTPs in Beijing indicated that the concentrations of particles (<2 μm) ranged from 1233 to 6533 per m^38^. 46.36 μg/m^3^ of SO_4_^2−^, 21.51 μg/m^3^ of NO_3_^−^, 19.76 μg/m^3^ of NH_4_^+^, and Mg^2+^, Cl^−^, K^+^, Na^+^, and Fe^2+^ were detected in the air surrounding the aerosol source^11^. Cl^−^, NO_3_^−^, SO_4_^2−^, and NH_4_^+^ were the main ions in the bioaerosols. Because of their small size and light weight, particles are easily carried by wind and dispersed over considerable distances. Previous studies have showed that the formation of SO_4_^2−^ and NO_3_^−^ increased during haze days in Beijing^12^. Water-soluble inorganic ions, such as NO_3_^−^, SO_4_^2−^_,_ and NH_4_^+^, are considered important contributors to visibility impairment^13,14^. It is well recognized that NO_3_^−^, SO_4_^2−^, and NH_4_^+^ play an important role in the formation of haze^15^.

In addition, chemicals, such as inorganic particles, soluble ions, and organic matter, may provide a suitable microenvironment for the survival of airborne microorganisms in the air and may contribute to urban smog. However, studies of the chemical components that accompany microorganisms in airborne particles released from WWTPs remain limited, particularly in China. By the end of 2016, over 4000 WWTPs have been constructed in China to meet the increasing treatment demand. The total treatment capacity was 170.79 million tons/d^16^. Nearly 40% of the WWTPs have implemented the anaerobic-anoxic-oxic (A^2^/O) treatment process or the sequencing batch reactor (SBR) treatment process, because of their economy and efficiency in wastewater purification. Injection of compressed air via submerged diffusers is widely applied to supply oxygen in aeration tanks.

This study was conducted to investigate microbes and chemicals in bioaerosols emitted from biochemical reaction tanks (BRTs) of six WWTPs in China. The selected WWTPs were in the Yangtze River and Zhujiang deltas and the Jing-Jin-Ji region. A scanning electron microscope (SEM) was used to observe the morphology of the particles in the bioaerosols. Factors that influence the survival of the bioaerosols in the air were analysed. The sources of the bioaerosols were identified via the SourceTracker method. Experiments were also conducted to explore the mechanism of bioaerosol formation during the aeration process. The objectives of this study were to (1) investigate the chemical components of the bioaerosols released from WWTPs in different regions, (2) analyse the relationship between the bioaerosol emission levels and meteorological parameters, and (3) explore the mechanism of bioaerosol generation during the aeration process in the BRTs. This work may provide the basis for the mitigation and control of bioaerosol emissions from WWTPs.

## Results

### Particle observation

Particles of interest and their aggregates in the bioaerosols were observed using a SEM^17^. The main component of the blank membranes (BMs) was SiO_2_ because glass fibre membranes were used as the media for the deposition of the particles. In the SEM images, SiO_2_ appeared to have a staggered mesh structure and, therefore, the membranes could intercept particles (see Supplementary Fig. S1). A wide variety of particles, of different physical appearances such as shape and size, were scattered amorphously on the membrane surfaces. Most of the particles observed were smaller than 50 μm. Such particles can be classified as either fine particles or coarse particles. Fine particles are those smaller than 2.5 μm, while coarse particles are in a range of 2. 5 μm to 10 μm^17,18^. Inhalation of particles smaller than 10 μm has harmful health effects.

The distribution of the particle sizes in the samples was related to the height of the sampling points. Sampling points were located at 0.1 m (0.1 WS), 1.5 m (1.5 WS), and 3.0 m (3.0 WS) above the water surface. The concentrations of particles were calculated using equation (1). Approximately 4891 particles per m^3^ of air were found in the samples collected from 0.1 WS. When the sampling height increased to 1.5 m and 3.0 m in the vertical direction, 3074 (for 1.5 WS) and 2152 (for 3.0 WS) particles per m^3^ of air were clustered on the membranes (Table 1). Particle concentration in the air decreases with an increase in the sampling height. The concentration of fine particles in the air surrounding the BRTs decreased accordingly. However, the percentages of fine particles presented an obvious trend of rising, with 59.46% detected in 0.1 WS, 66.35% found in 1.5 WS and 75.62% collected in 3.0 WS, respectively. After entering the surrounding air during the aeration process, large particles settle under the force of gravity while the smaller particles remain suspended in the air. In addition, the smaller lighter particles may disperse further than those that have heavier aggregate structures.

**Table 1:** Percentage and number of particles at different sampling heights.

	Percentage of coarse particles (2.5 μm–10 μm)	Percentage of fine particles (<2.5 μm)	Average total number (m^−3^)
0.1 WS	40.54%	59.46%	4891
1.5 WS	33.65%	66.35%	3074
3.0 WS	24.38%	75.62%	2152

(0.1 WS: Sampling point 0.1 m above the water surface; 1.5 WS: Sampling point 1.5 m above the water surface; 3.0 WS: Sampling point 3.0 m above the water surface).

Energy dispersive X-ray spectroscopy (EDX) analysis provided information on the elemental composition of particles (Table 2). Samples collected from BMs were also analysed to check the background elements. The main elements present in the BMs were Si and O, indicating that SiO_2_ was the main constituent of the membranes. For membranes with deposited particles, C, O, Si, S, Ca, Na, Al, Fe, and Mg were present. In addition, to C and O, the major elements were Si, Ca, Al, and Na which constituted 70.2% of the total elements. The main chemical compounds in the deposited particles were CaCO_3,_ Fe_2_O_3_, and NaCl. Fe_2_O_3_ was dominant in samples collected from 0.1 WS while CaSO_4_ was dominant in samples collected from 3.0 WS.

**Table 2:** Elements on the membranes.

Element (%)	Empty membranes	particles on sampled membrane (0.1 WS)	particles on sampled membranes (1.5 WS)	particles on sampled membranes (3.0 WS)
O	66.54	28.2	29.01	44.41
C	12.58	60.81	62.61	48.23
Si	11.52	3.57	2.81	2.54
Na	5.24	1.14	1.16	1.24
Al	1.4	0.91	0.76	0.68
Ca	0.64	1.05	1.93	2.07
Fe	0	1.3	0	0
Mg	0	0.14	0.18	0.09
S	0	0	0.18	0.57
Others	2.08	2.88	1.36	0.17
Total	100	100	100	100

(0.1 WS: Sampling point 0.1 m above the water surface; 1.5 WS: Sampling point 1.5 m above the water surface; 3.0 WS: Sampling point 3.0 m above the water surface).

### Bioaerosol microbial characteristics

**Figure 1:**
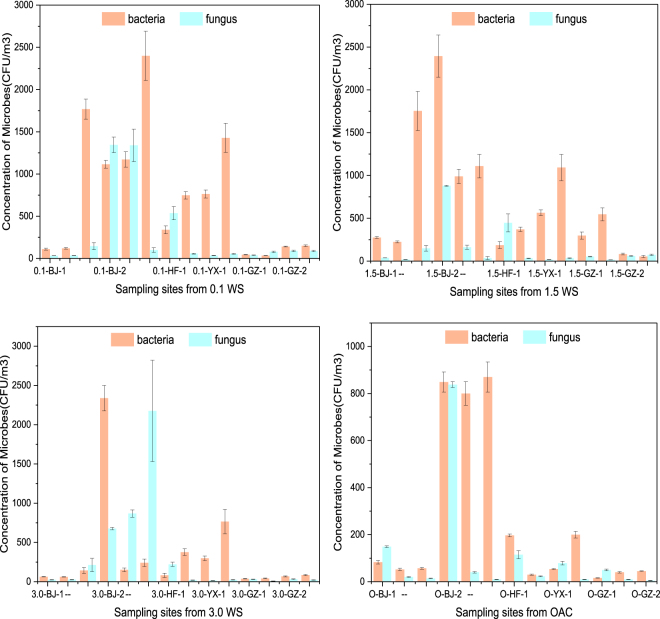
Airborne bacterial and fungal concentration (with standard deviation) at six WWTPs (CFU/m^3^). (WWTP: municipal wastewater treatment plants; The sample size was nine for each error bar).

The highest concentrations of bacteria and fungi were 2394 CFU/m^3^ and 879 CFU/m^3^, respectively, which were observed at the sampling point 1.5 WS (Fig. 1). The average concentration of microorganisms in the air surrounding the BRTs was 842 CFU/m^3^, while the concentration of microorganisms in the air surrounding the outdoor air control (OAC) was 334 CFU/m^3^, which is much lower than that in the air surrounding the BRTs. When the sampling height was increased from 0.1 m to 3.0 m, the average concentrations of bacteria and fungi decreased by 36.14%. There are numerous microbes that can degrade the microbes in the water of the BRTs. These microbes are transferred from water into the atmosphere during the aeration process. Therefore, bacteria and fungi are detected in the air surrounding the BRTs.

Bacterial populations in bioaerosols demonstrated that Moraxellaceae (11.17%), Pseudomonas (7.11%), Chroococcidiopsis (6.64%), Acinetobacter (3.17%), and Arcobacter (2.74%) were the main bacteria found in the air of the BRTs in the Jing-Jin-Ji region (see Supplementary Table S1). Meanwhile, Cyanobacteria_norank (80.46%) dominated the bioaerosols in the Yangtze River Delta, and Peptostreptococcaceae_incertae_sedis (13.79%), Mycobacterium (4.75%), and Sphingobacteriaceae (4.47%) were dominant in the surrounding air of the BRTs in the Zhujiang Delta. Results obtained in this study showed that bacterial populations in bioaerosols presented significant regional disparity. Among the identified bacterial species, some species, e.g. Bacteroides sp., Aeromonas, Arcobacter sp., and Flavobacterium sp., are common potential pathogens^18–20^ (see Supplementary Table S1). They have been isolated and identified from WWTPs in previous studies^21^. Pathogenic fungi, such as *Penicillium* and *Aspergillus*, can be distinguished by growing them in Petri dishes on appropriate media. These fungal genera produce harmful fungal toxins, such as aflatoxin B1, which is carcinogenic. Therefore, the transfer of such pathogenic microorganisms into the air might threaten the health of on-site workers of WWTPs^22^.

### Bioaerosol chemical components

#### Total suspended particles

Concentrations of total suspended particles (TSPs) in air samples collected from that surrounding the OAC were 91.93 µg/m^3^ for Beijing, 87.24 µg/m^3^ for Hefei, 68.90 µg/m^3^ for Yixing, and 69.42 µg/m^3^ for Guangzhou (see Supplementary Table S2). The BRT constitutes the aerobic wastewater treatment stage, in which oxygen is supplied by submerged diffuser. More particles were detected in the air samples collected during this stage than those sampled from the OAC. The average concentrations of TSPs were 80.66 µg/m^3^ (for BJ-1), 234.48 µg/m^3^ (for BJ-2), 115.71 µg/m^3^ (for HF-1), 138.90 µg/m^3^ (for YX-1), 99.83 µg/m^3^ (for GZ-1), and 81.87 µg/m^3^ (for GZ-2) (see Supplementary Table S2).

The range of TSP concentrations was between 59.05 µg/m^3^ and 324.88 µg/m^3^. The maximal TSP concentration was detected at BJ-2. The air samples collected from the WWTPs in Guangzhou showed the lowest TSP concentrations.

Beijing is in North China, Hefei and Yixing are in the Yangtze River Delta, and Guangzhou is in the Pearl River Delta. The population, lifestyle, degree of industrialization, geography, and climate of these regions differ. Therefore, there are regional disparities in the TSP concentrations in the air. In addition, factors such as water quality, treatment capacity, treatment process, and aeration rate could also contribute to a variation in air TSP concentrations. The water quality, treatment capacity, and meteorological conditions of each WWTP are described in Supplementary Tables S3 and S4.

The concentration of TSPs changes with a change in the sampling height. The average concentration of TSPs decreased from 223.95 µg/m^3^ to 137.19 µg/m^3^ when the sampling height increased from 0.1 m to 3.0 m.

The correlation between the air TSP concentration and the suspended solid (SS) concentration in water was analysed by Pearson correlation coefficient (see Supplementary Table S5). The coefficient between the TSP concentration of samples from 0.1 WS and the SS concentration in water was 0.91 (p < 0.05), which showed that the increase in the SS concentration in water is a vital factor in the increase in TSP concentration in air. The SS concentrations in the water of the BRTs in Beijing were the highest, which is among the reasons for the TSP concentrations being the highest in Beijing (see Supplementary Table S2).

#### Insoluble compounds in bioaerosols

TSPs comprise both soluble compounds (SCs) and insoluble compounds (ISCs). The percentage composition of ISCs in TSPs was 2.51–58.62% in Beijing, 3.32–10.48% in Hefei, 12.14–65.48% in Yixing, and 5.47–23.05% in Guangzhou (Table 3). The ISC composition percentage in TSPs varied for each WWTP. The maximal concentration of ISCs was detected at BJ-2 (148.34 μg/m^3^) which uses the A^2^/O process. This concentration is 63.39 times that of the minimal ISCs concentration (2.34 μg/m^3^). This is probably because the SS concentration in the water of BJ-2 was the highest at 361 mg/L (see Supplementary Table S2).

**Table 3:** Contents of SCs and ISCs in the sampling points at six WWTPs.

Sampling points	Sampling date	0.1 WS		1.5 WS		3.0 WS	
		SCs	ISc	SCs	ISc	SCs	ISc
BJ-1	Oct 23	161.65	95.75	83.21	6.44	78.14	11.26
	Mar 7	158.81	127.92	46.00	13.05	10.33	32.54
	Jun 24	160.79	94.15	90.95	2.34	142.01	45.81
BJ-2	Oct 24	216.04	191.39	197.22	8.50	131.80	65.33
	Mar 21	179.90	78.14	176.54	148.34	74.14	34.66
	Jun 29	145.10	328.60	71.52	101.31	76.24	114.33
HF-1	Apr 11	160.10	50.38	143.05	16.75	178.74	60.32
	Aug 1	121.35	8.21	69.24	2.38	36.09	36.41
YX-1	Apr 14	197.75	29.33	177.27	24.49	156.68	45.08
	Aug 2	52.48	74.32	26.25	49.80	28.38	84.44
GZ-1	Jan 21	75.88	13.15	60.39	9.06	87.91	37.18
	Sep 15	46.30	102.67	103.63	26.58	59.26	44.74
GZ-2	Jan 22	100.67	42.86	53.26	15.96	90.57	7.39
	Sep 16	83.78	37.87	89.35	5.17	71.87	79.02

(SCs: soluble compounds; ISCs: insoluble compounds).

#### Soluble compounds in bioaerosols

The majority of particles in the bioaerosols were SCs, accounting for approximately 79.03% of the total particles. The main components of bioaerosols were SCs such as organics and water-soluble ions.

**Figure 2:**
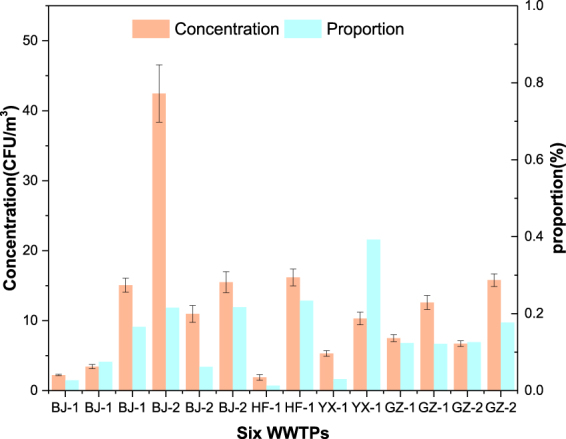
Concentration of TOC (with standard deviation) and the concentration of that in SCs at each WWTP. (TOC: total organic carbon; SCs: soluble compounds; The sample size was nine for each error bar).

**Total organic carbon:** The concentration of total organic carbon (TOC) in the water of each WWTP ranged from 23.28 mg/L to 160.00 mg/L. TOC comprises organics and secretions of microorganisms. Therefore, organic carbon could be detected in the particles released into the air. The concentrations of TOC and its proportion in SCs are shown in Fig. 2. The concentrations of TOC determined from the sampling points were in a range of from 2.23–42.47 µg/m^3^ in Beijing, 1.89–16.17 µg/m^3^ in Hefei, 5.31–10.30 µg/m^3^ in Yixing, and 6.71–15.79 µg/m^3^ in Guangzhou.

**Figure 3:**
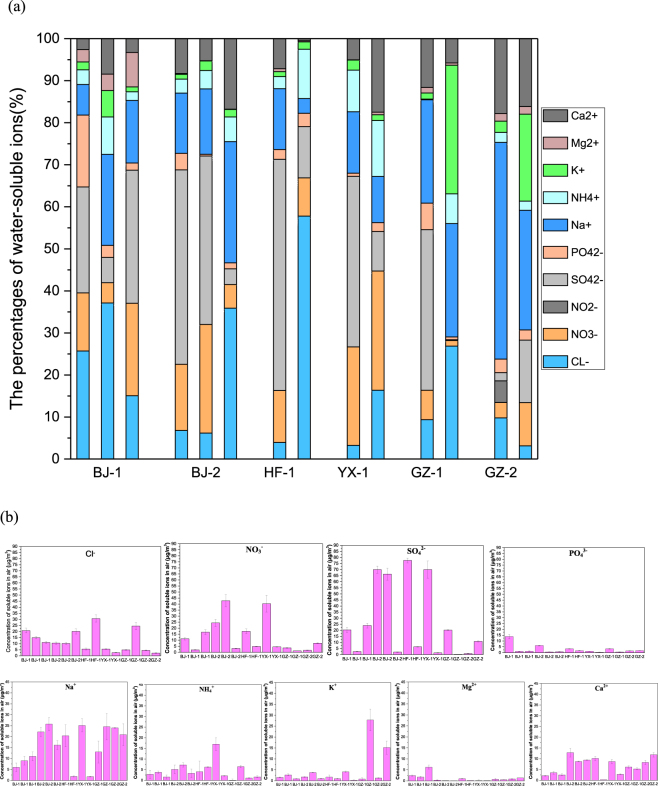
WSIs mass concentration in bioaerosols at each sampling points ((**a**) the percentage of water-soluble ions; (**b**) the concentration of different ions (with standard deviation)). (WSIs: water-soluble ions; The sample size was nine for each error bar).

**Ions:** Five cations (NH_4_^+^, Ca^2+^, K^+^, Mg^2+^, and Na^**+**^) and five anions (SO_4_^2−^, NO_3_^−^, Cl^−^, PO_4_^3−^, and NO_2_^−^) were detected in the air samples from the six WWTPs. Figure 3 shows the variations in the concentrations of water-soluble ions (WSIs) in the bioaerosols during the observational period. The total concentrations of WSIs were 97.12 µg/m^3^ in Heifei, 93.96 µg/m^3^ in Yixing, 95.96 µg/m^3^ in Beijing, and 66.01 µg/m^3^ in Guangzhou showing regional variation. The cation with the highest concentration was Na^+^ with a mean concentration of 15.07 µg/m^3^ in Beijing, 11.14 µg/m^3^ in Hefei, 13.44 µg/m^3^ in Yixing, and 20.62 µg/m^3^ in Guangzhou. Na^+^ accounts for 17.08% (for Beijing), 8.98% (for Hefei), 12.79% (for Yixing), and 32.88% (for Guangzhou) of the total ion concentration. The cation with the second highest concentration was Ca^2+^, accounting for 7.44% (for Beijing), 3.85% (for Hefei), 11.28% (for Yixing), and 12.84% (for Guangzhou) of the total ion concentration. The anion with the highest concentration was SO_4_^2−^, accounting for 25.47% (for Beijing), 33.58% (for Hefei), 24.98% (for Yixing), and 13.80% (for Guangzhou) of the total ion concentration. The anion with the second highest concentration was NO_3_^−^ with concentrations in a range of from 1.20 µg/m^3^ to 42.80 µg/m^3^. The major soluble chemical substances in the bioaerosols were identified as NaNO_3_, Ca (NO_3_)_2_, CaSO_4_, and Na_2_SO_4_. The bioaerosol elemental composition was similar to that of the wastewater. Na^+^, Ca^2+^, SO_4_^2−^, and NO_3_^−^ were the four dominants in the water collected from the six WWTPs (Fig. 3), which probably contributed to the high concentrations of these ions in the bioaerosols.

SO_4_^2−^ was the main soluble substance. A study investigating the cause of atmospheric haze showed that the formation of SO_4_^2−^ in the atmosphere is a decisive factor, to some degree, in haze formation in urban cities^15^. Previous studies have showed that the formation of SO_4_^2−^ and NO_3_^−^ accelerated during haze days in Beijing^12^. The chemicals emitted from WWTPs may disperse in the air, which could be conducive to haze formation in urban cities.

## Discussion

### Generation sources

**Figure 4:**
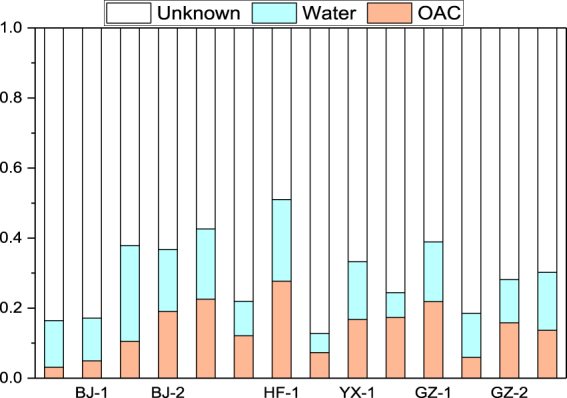
Source of bioaerosols.

The sources of bioaerosols generated by the six WWTPs are shown in Fig. 4. For the BRTs constructed in the WWTPs of Hefei and Yixing, the chemicals detected (e.g. TSP and WSIs) in the bioaerosols at the water surface were mainly chemicals that were originally present in the wastewater (23.34%) and OAC (27.69%), while the sources of the other chemicals were unknown (48.97%). The unknown sources were likely the surrounding soil and plants. The source proportion of bioaerosols from wastewater, OAC, and unknown sources was 16.73%, 12.05%, and 71.22%, respectively, for the BRTs of the WWTPs in Beijing, and 14.62%, 14.33%, and 71.05%, respectively, for the BRTs of the WWTPs in Guangzhou. Wastewater and outdoor air were the main sources of the chemicals detected in the bioaerosols in the air surrounding the BRTs. In the present study, all the BRTs of the WWTPs considered for bioaerosol investigation were outdoors. The compositions of bioaerosols in the air surrounding the BRTs were affected by the components in external atmosphere, particularly in air from the upwind direction. The OAC generated 14.19% of the bioaerosols.

On average, 15.09% of the bioaerosols found in the sampling point 0.1 WS were generated from wastewater. The BRT constitutes the aerobic wastewater treatment stage. Aeration facilities were built in the BRTs to provide oxygen (or air) to promote microbial activity that reduces the pollutants in the wastewater. During aeration, bubbles rise from the bottom of the BRT to the water surface. The difference between the bubble internal pressure and external pressure caused the bubbles to burst at the water surface and small water droplets formed. These droplets catapulted and escaped into the air. The activated sludge, inorganic particles, and soluble chemicals in the droplets were aerosolised and released into the atmosphere. Therefore, microorganisms and chemicals components that are initially present in water can be detected in bioaerosols.

At sampling point 0.1 WS, most of the bioaerosols were generated from water indicating that the chemicals detected in the bioaerosols were those that were originally present in the water. With an increase in the height of the sampling point, the proportion of chemicals in the bioaerosols originating from water decreased from 16.73% (for 0.1 WS) to 5.36% (for 3.0 WS) in the BRTs of the WWTPs in Beijing (see Supplementary Table S6). A similar phenomenon was observed in the WWTPs in Hefei, Yixing, and Guangzhou.

### Mechanism

**Figure 5:**
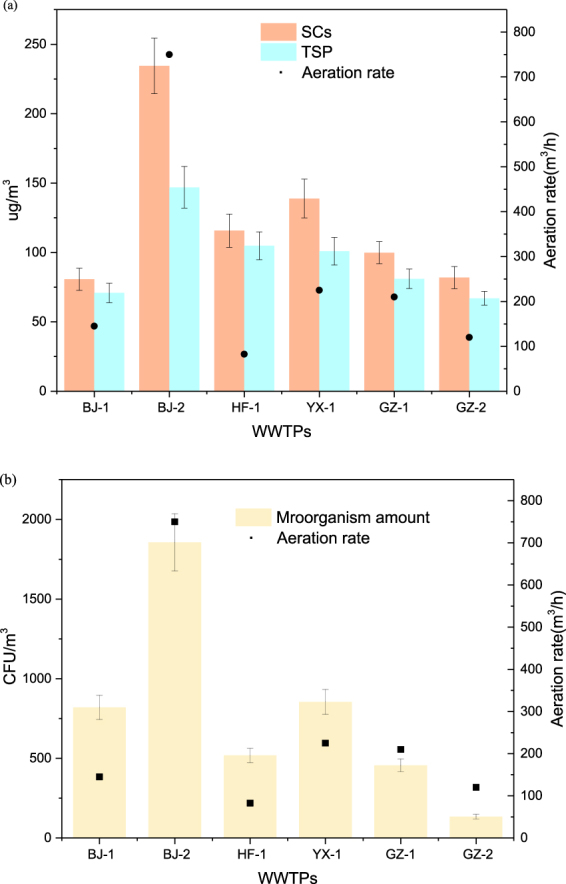
(**a**) Aeration rate of BRTs and variability in SCs and TSP concentration (with standard deviation) at each sampling point. (**b**) Aeration rate of BRTs and variability of airborne bacteria concentration (with standard deviation) at each sampling point.

The aeration rate in the BRT will influence the generation of bioaerosols and the concentrations of airborne bacteria and chemicals in the surrounding air. As schematised in Fig. 5b, 854 CFU/m^3^ of airborne bacteria, 138.90 μg/m^3^ of TSPs, and 100.88 μg/m^3^ of SCs were present in the surrounding air when the aeration rate exceeded 220 m^3^/h. There was a positive correlation between the aeration rate and the concentrations of airborne bacteria and chemicals in the surrounding air. This indicates that higher concentrations of airborne bacteria and chemicals will be emitted into the surrounding air when the aeration rate is increased.

In the present study, a submerged aeration mode was adopted at all six WWTPs. Aeration facilities produce bubbles at the bottom of the BRT during the aeration process. Bubbles rise via the water and eventually burst when they reach the water surface, projecting a large amount of film drops into the air. The birth of film drops originating from bubbles bursting on the water surface can be described by the Rayleigh instability theory^23^. The process of film drop generation can be divided into 3 stages. During stage 1, a film cap, which could be considered as a thin curved liquid film, is formed. During stage 2, a collapsing bubble cavity appears at the film cap, after it becomes increasingly thinner and propagates from there collecting the film’s mass into a toroidal ring as it progresses. This process is enabled because the surface tension of the toroidal ring provides the force required to sustain the centripetal accelerations. During stage 3, the surface tension is insufficient to maintain the centripetal accelerations at the toroidal ring and film drops are created^23–25^. Hundreds of film drops can be created per bubble burst. The cap disintegration process consists of the disintegration of the thin liquid film separating the collapsing bubble cavity from the atmosphere.

Bubble bursting tests were conducted in a laboratory to investigate the effect of the aeration rate on the generation and distribution of film drop generation. The results showed that most of the film drops that are produced because of the bursting of bubbles have diameters less than 2 mm. When the aeration rate was 0.2 L/min for 1 min, approximately 11 bubbles burst, producing 1290 film drops. The average number of film drops produced increased to 2351 per minute when the aeration rate increased from 0.2 L/min to 0.4 L/min. When the aeration rate increases, more bubbles will burst during the same aeration time, which results in the production of more film drops (see Supplementary Table S7).

Spatial variations in the concentrations of chemicals and microorganisms in the bioaerosols were observed as the aeration rate increased. In the air surrounding the WWTPs, the concentration of microorganisms and chemicals was 95 CFU/m^3^ and 22.04 μg/m^3^, respectively, at 0.1 WS with an aeration rate of 0.2 L/min. When the aeration rate increased to 0.6 L/min, which was three times that of the former aeration rate, the concentration of chemicals and microorganisms also increased by 3.97 and 2.81 times, respectively. These results illustrate that a higher water aeration rate might increase the concentrations of chemicals and microorganisms in bioaerosols (see Supplementary Table S8).

When the number of bubbles that burst increases, the variations in the concentration of film drops were more obvious in both the horizontal distribution and vertical distribution (see Supplementary Fig. S2). The vertical distribution of film drops showed a higher concentration of 5273 in the lower part (0–0.09 m) and a lower concentration of 1625 in the upper part (0.09–0.18 m) with 55 bubbles bursting (see Supplementary Table S7). Whereas, the horizontal distribution of film drops appeared to be more similar than the vertical distribution of film drops. When the sampling height increased from 0.01 m to 0.1 m, the concentration of chemicals and microorganisms in bioaerosols decreased by 92 CFU/m^3^ and 15.09 μg/m^3^, respectively (see Supplementary Table S8). As the height increases, the number of particles decreases.

### Survival factors

The distribution of bioaerosols in the air was influenced by factors such as wind diffusion, large particle sedimentation, and wind dilution. Particles will spontaneously diffuse from high concentration areas to low concentration areas showing spatial distribution characteristics. A previous study has identified the trend in the dispersion of particles from higher concentration areas to lower concentration areas in the air in urban areas^26^. When the sampling heights increased from 0.1 m to 3.0 m, the concentrations of bacteria and fungi decreased by 23.71% (Fig. 1), while the concentration of chemicals, TSPs, and TOC decreased by 36.86%, 38.74%, and 10.72%, respectively (see Supplementary Tables S2 and S9–S12). Previous studies of the emission of bioaerosols from WWTPs have reported similar findings and showed that bioaerosols can also be influenced by wind direction and distance from their source^27^.

Particle sedimentation, particularly large particles, was identified as another reason for the variation in the vertical distribution of bioaerosols in the atmosphere. Because of gravity, large particles in bioaerosols may settle and fall back into the water. SEM images have showed that the number and percentage of coarse particles in samples from 3.0 WS was much lower than that in the samples from 0.1 WS, indicating that coarse particles in the bioaerosols emitted from the BRTs readily settled.

The decrease in the bioaerosol concentration in the air was because of diffusion by wind as the BRTs were outdoors. During the sampling period, the wind speed in Beijing, Heifei, Yixing, and Guangzhou was 2.98 m/s, 2.11 m/s, 1.56 m/s, and 0.33 m/s, respectively (see Supplementary Table S4). The wind may carry light particles, and therefore reduce the particle concentration in the air.

**Figure 6:**
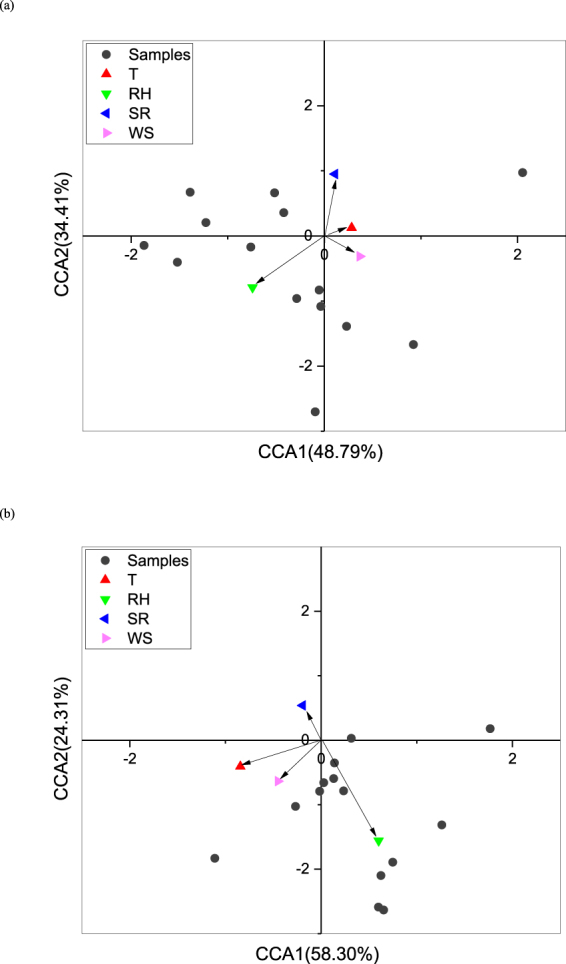
(**a**) Canonical correlation analysis of ions with respect to the relative abundances of meteorological conditions. (**b**) Canonical correlation analysis of microorganism with respect to the relative abundances of meteorological conditions. (T: Temperature; RH: Relative humidity; SR: Solar radiation; WS: Wind Speed).

The results of a Canonical correlation analysis, which was conducted to predict the factors affecting the microbial population and the chemicals in bioaerosols, agree with the results of the analysis of the effects of wind on bioaerosols (Fig. 6)^28^.

In addition to WS, other meteorological factors such as temperature (T), solar radiation (SR), and relative humidity (RH) were also predicted as factors that influence bioaerosols using Canonical correlation analysis in the present study. For the microbial population of bioaerosols, RH and T had a positive influence while WS and SR had a negative influence. The chemicals in bioaerosols had a positive relationship with RH and a negative relation with WS. The sedimentation of large particles and dilution and diffusion by wind led to spatial variations in the distribution of aerosols in the air.

### Spatial variations

Sampling points at the BRTs were established in a vertical direction, 0.1 m, 1.5 m and 3.0 m above the water surface, to investigate the characteristics of bioaerosols suspended at greater heights. Bioaerosols collected in the sampling points of 0.1 WS were those formed after bubble burst. The dispersion of bioaerosols in a vertical direction was investigated at the sampling points of 1.5 WS and 3.0 WS. The highest concentration of bioaerosols emerged at 0.1 WS, where the total average number of particles was 4891 m^−3^. When the height of the sampling point increased from 0.1 m to 1.5 m. The percentage of fine particles in the total particles increased from 59.46% to 66.35%. The total number of fine particles was still 3074 m^−3^ at 1.5 m and the concentration was approximately 25 µg/m^3^ (estimated by PM2.5). Generally, a height of 1.5 m is regarded as the human respiratory height. Fine particles are more harmful to human health than those that are coarse in size because the former enter the human body more easily than do coarse particles. Fine particles are directly inhaled and adhere to the respiratory tract and alveolar region^29^. Previous study has reported that an annual exposure to 5 µg/m^3^ of fine particles was linked to a 13% increased risk of heart attacks^30^. Therefore, effective measures should be adopted to protect on-site workers from the damage of the fine particles. There were 2152 m^−3^ particles found in the air 3.0 m above the water surface. Over 70% were fine particles. Because of their small size and light weight, fine particles are easily carried by wind and dispersed over considerable distances. They may be inhaled and harm residents living in an area where they settle.

## Materials and Methods

### Overview of the wastewater treatment plants and sample collection process

Bioaerosol emissions from six WWTPs in Beijing (BJ-1 and BJ-2), Hefei (HF-1), Yixing (YX-1), and Guangzhou (GZ-1 and GZ-2) were investigated (see Supplementary Fig. S3). Sampling points were established near the BRTs, aeration basin, and aerobic biochemical pool. A submerged aeration mode was adopted in all six WWTPs. Samples were collected at heights of 0.1 m, 1.5 m, and 3.0 m above the water surface, which were termed 0.1 WS, 1.5 WS, and 3.0 WS, respectively, in this study. Three replicates were taken consecutively at every sampling point. OAC points were designed at the boundary of the WWTPs, 80 m upwind of the BRTs. Samples for OAC were placed on a platform 1.5 m above the ground concentration.

### Experimental process

A lab-scale bubble-generating device, measuring 600 (length) × 300 (width) × 500 (height) mm^3^ and made of polyvinyl chloride, was set up to investigate the process of bioaerosol generation (see Supplementary Fig. S4). A perforated tube (10 mm in diameter) with one bubble hole (1.0 mm in diameter) serving as the nozzle was fixed at the bottom of the aeration tank to form bubbles. The tank contained a certain volume of wastewater coloured with black ink to observe the coloured bubbles generated by the aeration. The liquid level of the tank was set at 5 mm, just above the nozzle, such that the bubbles remained attached to the nozzle during the process of production, growing in size, and then bursting out of the nozzle. This ensured neutral buoyancy of bubbles. Rice paper, with a thickness of 0.08 mm and a density of 410.9 kg/m^3^, was used to collect droplets. This was completed to clearly observe the initial appearance and distribution of the droplets.

After the collection of droplets, removed papers were flattened and scanned to convert the droplet information into images. The images were processed using MATLAB programs to obtain information regarding the number and spatial location of the droplets. The ion and microbe concentrations were also measured.

### Sample collection and preparation

Medium-flow samplers (TH-150, Wuhan, China) were used to collect TSPs in the air surrounding each WWTP. A medium-flow sampler is a type of impingement sampler; it is portable, small, and is used for highly accurate monitoring. Glass fibre membranes were used as filter media for sample deposition and were also used for chemical and microbial analyses and particle observation. The membranes had a 99.90% particle rejection coefficient and were 90 mm in diameter. Before and after sampling, the membranes were dried in a CaCl_2_ desiccator for 48 h and weighed for gravimetric determination of TSPs using an electronic balance with a detection limit of 0.1 mg (AL204, Mettler Toledo, Columbus, OH, USA). The membrane holders were cleaned with 75% ethanol before use. The medium-flow samplers were used to collect samples continuously for 4 h at a flow rate of 100 L/min. After sampling, the membranes were stored in a bacteria-free environment at −20 °C.

### Analysis method

#### Particle observation

After sampling, the surface properties of the membranes were observed using an SEM (HITACHIS- 3000 N/EDX Inc., Japan) after treatment with acids or alkalis. A certain number (up to 100) of viewing fields were selected on each membrane, according to nine pairs of coordinates, for analysis using a systematic sampling design as completed in a previous study^31^. The magnification was adjusted to ensure that the visual properties of the particles including number, size, shape, type, and aggregation status were clearly visible. Viewing fields of each membrane were scanned at magnifications of 500×, 1000×, and 5000×.

The number concentrations of particles were calculated using equation (1) as follows:

1 $$c={\rm{\Delta }}n\times s/{\rm{\Delta }}s\times V$$

where, c is the number concentration of particles (per cm^3^), Δn denotes the average number of particle in each viewing field, Δs is the average area of each viewing field (mm^2^), s is the area of the membrane (mm^2^), and V denotes the volume of air that passes through the membrane (cm^3^).

The distribution of elements on the surface or in the pores of the carbon particles were determined using an SEM coupled with EDX.

#### Microbial analysis

Each sample was diluted into at least three gradients and plated on three media. Enumeration of viable microorganism-colony count was completed by inoculation in a lysogeny broth (LB) agar culture medium. The total DNA of the bacteria from samples was extracted using the Power Soil DNA Isolation Kit (Mo Bio Laboratories, Carlsbad, CA, USA). The hypervariable V3 and V4 regions of the bacterial 16 S rRNA gene sequences were amplified by primers of 338 F/806 R to Illumina MiSeq sequencing. Purified amplicons were pooled in equimolar and paired-end sequenced (2 × 300). High-throughput sequencing of the mixture of amplicons using the Illumina MiSeq platform was performed by Majorbio Bio-Pharm Technology Co., Ltd. (Shanghai, China) following the standard manufacturer’s instructions.

#### Chemical analysis

After sampling, each membrane was cut and the chemical components were extracted with 50 mL of ultra-pure water (18.2 MΩ, Millipore, Billerica, MA, USA) in an ultrasonic bath for 20 min at room temperature (25 °C). The extract was filtered through filters with a pore size of 0.22 μm. After extraction, the membranes were dried to obtain the insoluble substances, while the filtrate was used for an analysis of soluble matter. The filtrate was diluted 10 times for the analysis of soluble matter. Each sample was analysed three times.

A TOC analyser (TOC-V-CPH Shimadzu, Kyoto, Japan) was used to determine the TOC content in the samples. Concentrations of SO_4_^2−^, NO_3_^−^, Cl^−^, PO_4_^3−^, and NO_2_^−^ (anions) in each sample were determined using an ion chromatograph (ICS-3000, Dionex, Sunnyvale, CA, USA). Concentrations of NH_4_^+^, Ca^2+^, K^+^, Mg^2+^, and Na^+^ (cations) in each sample were determined using an ion chromatography system (IC plus 883, Metrohm, Herisau, Switzerland).

The chromatography system comprises a column oven, conductivity detector, manual injector, and chromatography workstation (Metrohm). The ion chromatography conditions were as follows: AS19 column and Metrosep C 4150/4.0 separation column; eluent: 20 mM NaOH (anions), 2.0 mM HNO_3_ (cations); column temperature: 30 °C; flow-rate: 1.0 mL/min; injection volume: 10 L. The detection limit of the method was less than 0.05 mg/L for both the anions and cations.

#### Statistical Analyses

Raw FASTQ files were de-multiplexed and quality-filtered using QIME (version 1.17). Paired-end reads were filtered first and high-quality sequences were clustered using Usearch (vsesion 7.1 http://drive5.com/uparse/) into operational taxonomic units (OTUs) identified with a cutoff of 97% identity. The Quantitative Insights into Microbial Ecology (QIME) program was adopted to construct representative sequences for clean reads of filtered OTUs. Taxonomy was assigned to each representative sequence using an RDP classifier with an 80% bootstrap confidence threshold. Representative bacterial sequences were assigned at different taxonomic levels (from phylum to genus) to the SILVA database (Release115 http://www.arb-silva.de). A rarefaction analysis based on Mothur V.1.21.1 was conducted to show the diversity indices, including the Chao and Shannon indices.

SourceTracker is a Markov-Chain Monte Carlo based method. It can be used to analyse sources and attributions in a specific environment. The efficacy of the SourceTracker software has been investigated by Christopher Staley and his group^32^. The initial library showed SourceTracker had a 91% accuracy in identifying the presence of source contamination using local sources^32^. SourceTracker was used in this study to analyse water and outdoor air attributions and profile potential sources of bioaerosols. Canonical correspondence analysis, Pearson correlation analysis, and SourceTracker analysis were conducted using the R software package (version 3.3.3).

## Electronic supplementary material

Chemicals and microbes in bioaerosols from reaction tanks of six wastewater treatment plants: survival factors, generation sources, and mechanisms
