# Supplementary material for: Chemicals and microbes in bioaerosols from reaction tanks of six wastewater treatment plants: survival factors, generation sources, and mechanisms
Source: Sci Rep. 2018 Jun 19;8:9362. doi: 10.1038/s41598-018-27652-2 (PMC6008454; doi:10.1038/s41598-018-27652-2)
Supplement: Supplementary file 1 — Chemicals and microbes in bioaerosols from reaction tanks of six wastewater treatment plants: survival factors, generation sources, and mechanisms [file 41598_2018_27652_MOESM1_ESM.pdf]

Chemicals and microbes in bioaerosols from reaction tanks of six wastewater treatment plants: survival factors, generation sources, and mechanisms

Yanjie Wang[1,2], Huachun Lan[3], Lin Li[1,2*], Kaixiong Yang[1,2], Jiuhui Qu[3], Junxin Liu[1,2]

wangyanjie_2008@126.com.cn, hclan@tsinghua.edu.cn, leel@rcees.ac.cn, yang_kaixiong@126.com, jhqu@tsinghua.edu.cn, jxliu@rcees.ac.cn

[1] State Key Laboratory of Environmental Aquatic Chemistry, Research Center for Eco-Environmental Sciences, Chinese Academy of Sciences, Beijing 100085, China.

[2] National Engineering Laboratory for VOCs Pollution Control Material & Technology, University of Chinese Academy of Sciences, Beijing 101408, P. R. China.

[3] School of Environment, Tsinghua University, Beijing 100084, China

[*]Corresponding author: Lin Li, Tel.: 86-10-62923543, E-mail: leel@rcees.ac.cn

Supplementary Figure S1. Overview of particles on membranes under 1000 and 5000 magnification times.

Supplementary Figure S2. The spatial distributions of drops.

Supplementary Figure S3. Schematic diagram of sampling point locations. ★: Sampling point.

Supplementary Figure S4. Bubble generation device schematic diagram.

Supplementary Figure S1. Overview of particles on membranes under 1000 and 5000 magnification times.

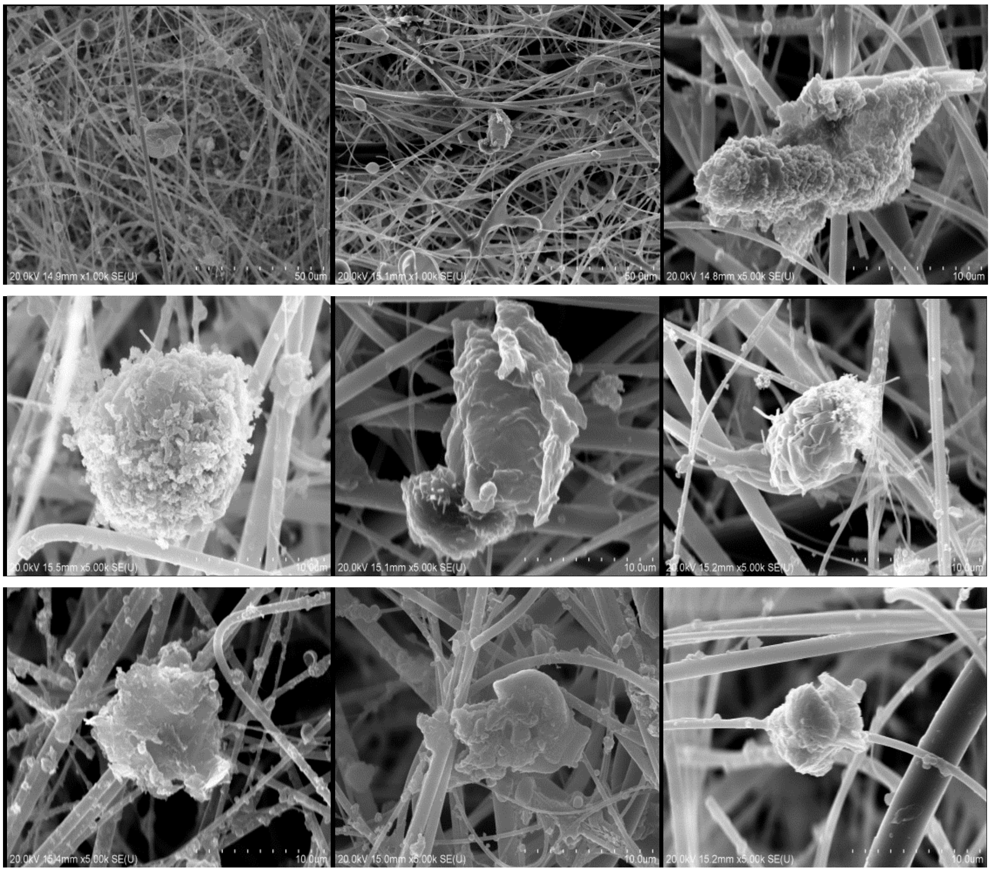

Supplementary Figure S2. The spatial distributions of drops.

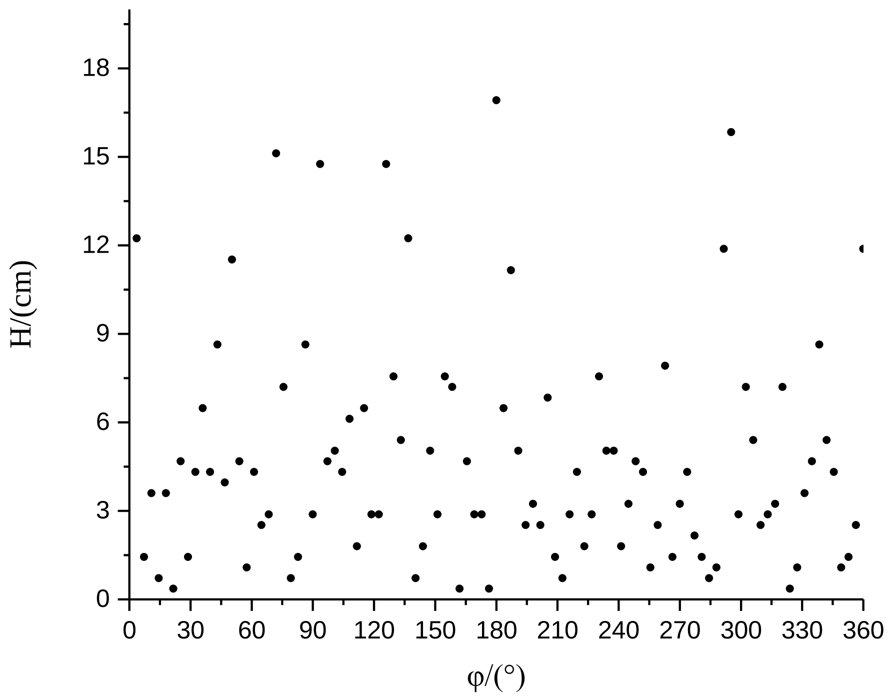

Supplementary Figure S3. Schematic diagram of sampling point locations. ★: Sampling point (Microsoft Office Visio 2016, https://products.office.com/en-us/home).

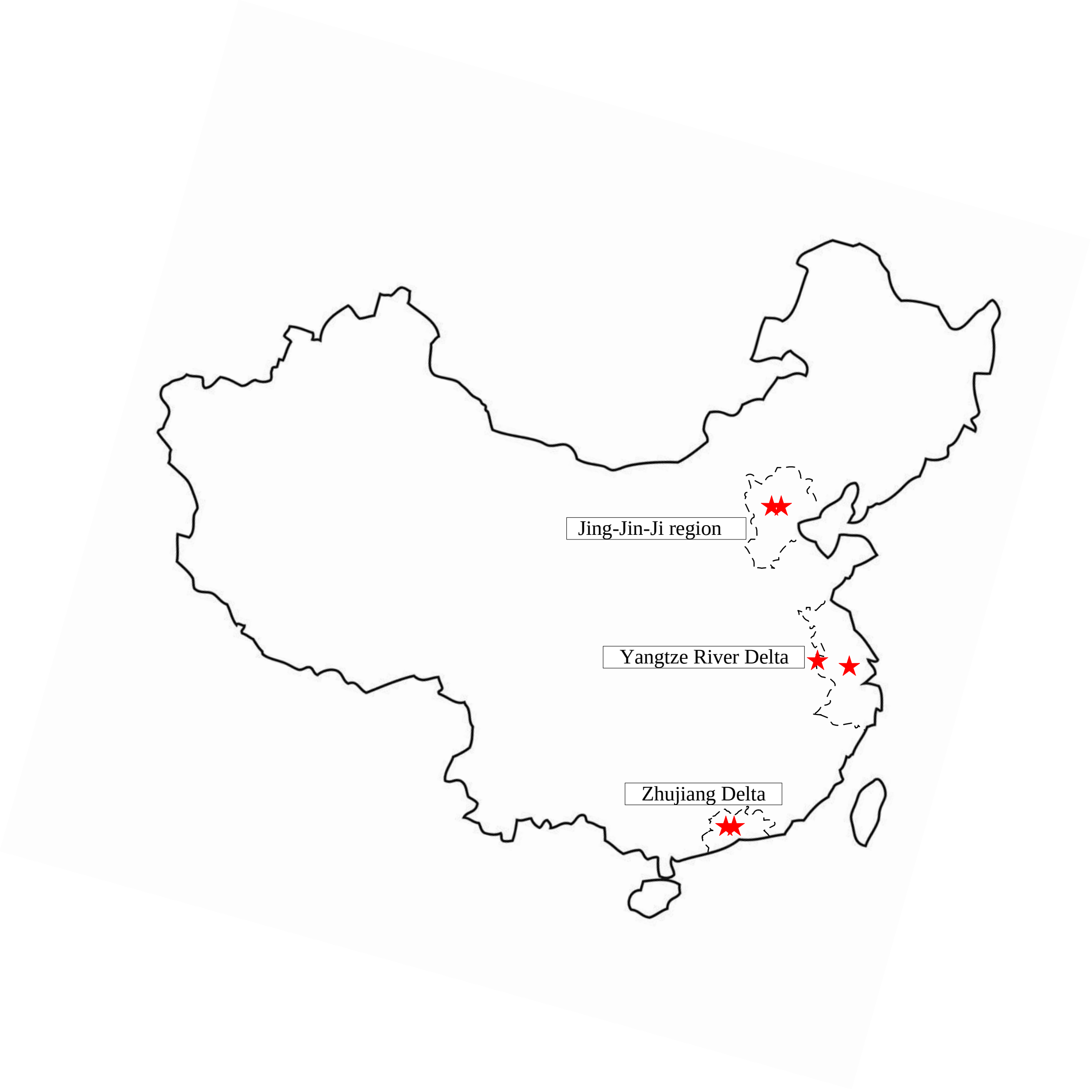

Supplementary Figure S4. Bubble generation device schematic diagram (Microsoft Office Visio 2016, https://products.office.com/en-us/home).

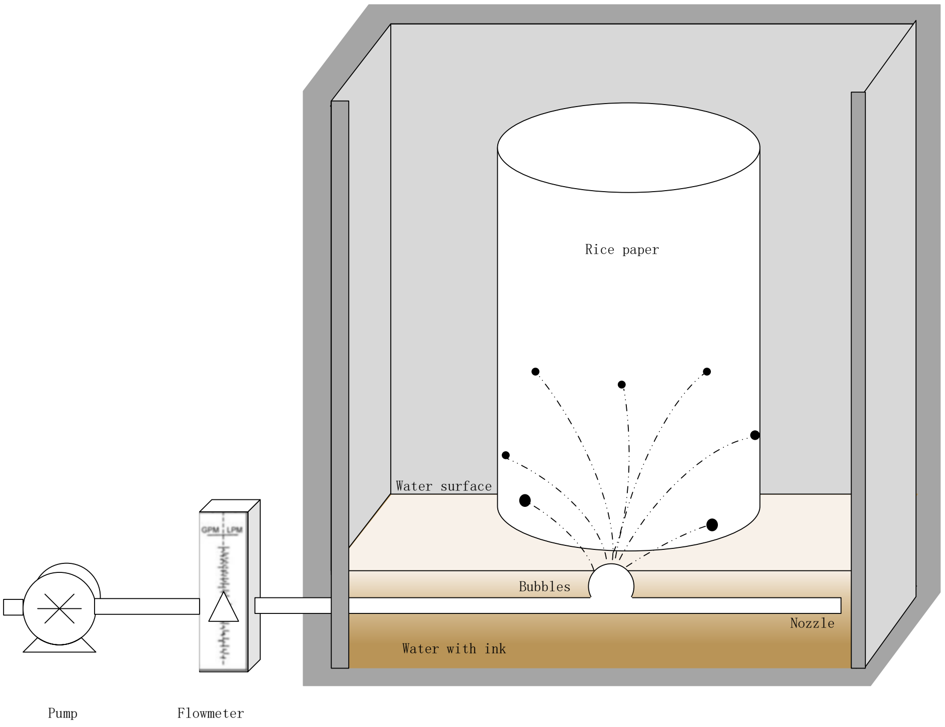

Supplementary Table S1. The microbial abundance in the bioaerosol samples of WWTPs from different regions.

Rank	Jing-Jin-Ji region		Yangtze River Delta				Zhujiang Delta	
	Beijing	Proportion	Hefei	Proportion	Yixing	Proportion	Guangzhou	Proportion
1	*Moraxellaceae_uncultured*	11.17%	*Cyanobacteria_norank*	80.46%	*Cyanobacteria_norank*	28.65%	*Peptostreptococcaceae_incertae_sedis*	13.79%
2	*Pseudomonas*	7.11%	*Moraxellaceae_uncultured*	3.68%	*Candidatus_Microthrix*	8.76%	*Mycobacterium*	4.75%
3	*Chroococcidiopsis*	6.64%	*Chroococcidiopsis*	2.97%	*Saccharibacteria_norank*	4.55%	*Sphingobacteriaceae_unclassified*	4.47%
4	*Acinetobacter*	3.17%	*Sphingomonas*	0.83%	*Romboutsia*	3.36%	*Moraxellaceae_uncultured*	3.86%
5	*Arcobacter*	2.74%	*Cyanobacteria_uncultured*	0.67%	*Chloroflexi_uncultured*	3.14%	*Mitochondria_norank*	3.45%
6	*Ralstonia*	2.61%	*Xanthomonadaceae_uncultured*	0.41%	*Saprospiraceae_uncultured*	3.08%	*Sphingomonadales_unclassified*	2.88%
7	*Neisseriaceae_uncultured*	2.39%	*Geodermatophilus*	0.37%	*Chroococcidiopsis*	2.73%	*Candidate_division_TM7_norank*	2.77%
8	*Escherichia-Shigella*	1.98%	*Hymenobacter*	0.35%	*Chitinophagaceae_uncultured*	1.62%	*Chitinophagaceae_uncultured*	2.58%
9	*Halomonas*	1.97%	*Methylobacterium*	0.33%	*Sphingobacteriales_unclassified*	1.28%	*Sphingomonas*	2.35%
10	*Cyanobacteria_norank*	1.55%	*Comamonadaceae_unclassified*	0.32%	*Comamonadaceae_unclassified*	1.27%	*Flexibacter*	1.75%

Supplementary Table S2. Total suspended particulates and suspended substance concentration in different sampling points from Municipal wastewater treatment plants. (TSPs: total suspended particulates; SS: suspended substance; 0.1WS: Sampling points located 0.1m above the water surface; 1.5WS: Sampling points located 1.5m above the water surface; 3.0WS: Sampling points located 3.0m above the water surface)

Sampling points	Sampling date	TSPs (μg/m^3)	TSPs (μg/m^3)	TSPs (μg/m^3)	TSPs (μg/m^3)	SS (mg/L)
		1.5WS	0.1 WS	3.0 WS	OAC	Wastewater
BJ-1	Oct 23	89.65	257.40	89.40	51.62	300
	Mar 7	59.05	286.73	42.87	39.74	320
	Jun 24	93.29	254.94	187.82	61.86	325
BJ-2	Oct 24	205.72	407.43	197.13	122.42	357
	Mar 21	324.88	258.04	108.80	188.62	361
	Jun 29	172.83	473.70	190.57	87.33	368
Average		157.57	323.04	136.10	91.93	339
HF-1	Apr 11	159.80	210.48	239.06	119.85	249
	Aug 1	71.62	129.56	72.50	54.63	180
Average		115.71	170.02	155.78	87.24	215
YX-1	Apr 14	201.76	227.08	201.76	92.71	271
	Aug 2	76.05	126.8	112.82	45.09	130
Average		138.91	176.94	157.29	68.90	201
GZ-1	Jan 21	69.45	89.03	125.09	90.47	134
	Sep 15	130.21	148.97	104.00	59.58	245
GZ-2	Jan 22	69.22	143.53	97.96	39.94	215
	Sep 16	94.52	121.65	150.89	87.70	197
Average		90.85	125.80	119.49	69.42	198

Supplementary Table S3. Municipal wastewater treatment plants description.

Sampling points		BJ-1	BJ-2	HF-1	YX-1	GZ-1	GZ-2
Location		Beijing		Hefei	Yixing	Guangzhou	
Sewage source		M-DS-IS	DS	DS	M-DS-IS	DS	DS
Capacity (×10^4 m^3/d)		3	50	5.5	7.5	8	15
Water quality	COD(mg/L)	527.12	500.42	225.59	250.65	400.77	260.51
	NH4-N(mg/L)	41.31	43.53	19.25	37.46	47.96	35.42
	TN(mg/L)	76.42	62.85	35.51	30.34	40.38	45.11
	TP(mg/L)	4.20	5.26	5.43	2.75	8.79	4.47
	TOC(mg/L)	156.14	113.97	109.57	115.84	160.11	67.36
	SS(mg/L)	300.25	357.27	249.68	271.42	134.34	214.21

Supplementary Table S4. Sampling time and meteorological conditions.(T: Temperature; RH: Relative humidity; SR: Solar radiation; WS: Wind Speed； DS: domestic sewage; IS: industrial sewage; M-DS-IS: Mixture of domestic sewage and industrial sewage)

Location	Sampling points	Sampling date	Meteorological conditions			
			T (^{0}C)	RH (%)	SR (W/m^2)	WS (m/s)
Beijing	BJ-1	Oct 23	19.00	18.00	420.00	0.56
		Mar 7	10.90	7.70	439.50	0.75
		Jun 24	36.90	31.50	216.16	11.00
	BJ-2	Oct 24	11.20	36.40	395.80	2.02
		Mar 21	17.20	23.20	434.00	0.50
		Jun 29	35.30	37.80	546.40	3.10
Heifei	HF-1	Apr 11	24.20	27.00	303.00	1.78
		Aug 1	37.20	47.40	585.60	2.44
Yixing	YX-1	Apr 14	25.50	44.00	519.00	1.38
		Aug 2	31.90	54.10	576.50	1.74
Guangzhou	GZ-1	Jan 21	19.80	78.90	120.00	0.89
		Sep 15	34.50	57.50	232.20	0.01
	GZ-1	Jan 22	19.90	54.50	300.00	0.02
		Sep 16	37.50	46.30	311.70	0.39

Supplementary Table S5. Pearson Correlation Coefficient of total suspended particulates in air with suspended substance concentration in different sampling points from wastewater treatment plants.

Sampling sits	Beijing	Hefei and Yixing	Guangzhou
0.1WS	0.828（p=0.042）	0.955（p=0.045）	0.953（p=0.047）
1.5WS	-0.329（p=0.525）	0.932（p=0.068）	0.765（p=0.235）
3.0WS	0.609（p=0.199）	0.803（p=0.197）	-0.366（p=0.634）

Supplementary Table S6. Source of bioaerosols in different sampling height.

		0.1WS			3.0WS		
	Sampling date	OAC	Water	Unknown	OAC	Water	Unknown
BJ-1	Oct 23	3.14%	13.30%	83.56%	18.20%	2.86%	78.94%
	Mar 7	4.95%	12.18%	82.87%	12.45%	12.93%	74.62%
	Jun 24	10.51%	27.34%	62.15%	13.34%	1.40%	85.26%
BJ-2	Oct 24	19.03%	17.71%	63.26%	14.77%	7.25%	77.98%
	Mar 21	22.57%	20.02%	57.41%	15.70%	3.11%	81.19%
	Jun 29	12.10%	9.83%	78.07%	19.81%	4.61%	75.58%
HF-1	Apr 11	27.69%	23.34%	48.97%	13.81%	6.71%	79.48%
	Aug 1	7.32%	5.41%	87.27%	11.68%	3.49%	84.83%
YX-1	Apr 14	16.74%	16.50%	66.76%	14.54%	9.99%	75.47%
	Aug 2	17.33%	7.09%	75.58%	21.42%	3.42%	75.16%
GZ-1	Jan 21	21.86%	17.06%	61.08%	20.64%	18.24%	61.12%
	Sep 15	5.95%	12.55%	81.50%	14.43%	8.93%	76.64%
GZ-1	Jan 22	15.81%	12.36%	71.83%	35.39%	15.62%	48.99%
	Sep 16	13.71%	16.51%	69.78%	14.05%	16.60%	69.35%

Supplementary Table S7. The number of drops.

Aeration rate (L/min)	Sampling time(min)	NBB	NFD	ANFD	$ANFD_{air}$	LP	UP
0.2	1	11	1290	117	6450	1032	258
0.2	5	55	6898	116	6898	5273	1625
0.4	1	29	3641	126	9102	2978	663
0.6	1	75	10173	136	16955	8795	1378

NBB: Number of bubble bursting; NFD: Number of film drop； ANFD: The average number of film drops per bubble bursting; $ANFD_{air}$: The average number of film drops/L_{air} ; LP: Lower part(0 m-0.09 m)； UP: Upper part(0.09 m-0.18 m)

Supplementary Table S8. The concentration of microorganism and chemicals from bubble generation device.

Aeration rate (L/min)	Calculational area	Bacteria (CFU/m^3)	CL (μg/m^3)	NO3 (μg/m^3)	NO2 (μg/m^3)	SO4 (μg/m^3)	PO4 (μg/m^3)	Total Chemicals (μg/m^3)
0.2	0-0.01m	95	3.41	15.01	0.47	2.48	0.66	22.03
	0.01-0.05m	20	2.73	13.00	0.25	1.99	0.25	18.22
	0.05-0.10m	3	1.18	4.64	0.09	1.03	0.00	6.94
	0.01-0.1m	7	1.64	7.28	0.13	1.32	0.07	10.44
0.4	0-0.01m	238	9.13	40.23	1.27	6.65	1.78	59.06
	0.01-0.05m	50	6.52	30.42	0.69	5.60	0.70	43.93
	0.05-0.10m	8	2.83	10.85	0.24	2.59	0.00	16.51
	0.01-0.1m	18	3.92	17.04	0.28	2.78	0.15	24.17
0.6	0-0.01m	371	10.23	57.13	2.19	11.44	3.06	84.05
	0.01-0.05m	77	8.19	43.19	1.22	9.86	1.23	63.69
	0.05-0.10m	12	3.55	15.41	0.37	4.04	0.00	23.37
	0.01-0.1m	28	4.92	24.20	0.51	3.58	0.23	33.44

Supplementary Table S9 The concentration of ions and TOC in aerosols at sampling points of 0.1WS (TOC: total organic carbon; 0.1WS: Sampling points located 0.1m above the water surface).

0.1WS	Cl^- (μg/m^3)	NO_3^- (μg/m^3)	NO_2^- (μg/m^3)	SO_4^{2-} (μg/m^3)	PO_4^{3-} (μg/m^3)	Na^+ (μg/m^3)	NH_4^+ (μg/m^3)	K^+ (μg/m^3)	Mg^{2+} (μg/m^3)	Ca^{2+} (μg/m^3)	TOC (μg/m^3)
BJ-1	34.00	0.00	0.00	77.09	0.00	21.18	0.00	2.74	0.25	16.92	9.47
BJ-1	22.30	2.08	0.00	54.28	0.00	20.55	0.00	1.36	0.87	15.67	41.70
BJ-1	30.07	20.50	20.50	41.33	0.00	17.18	0.00	1.42	0.36	13.85	15.58
BJ-2	24.30	28.00	0.00	80.67	4.20	23.02	10.95	2.60	0.19	24.91	17.20
BJ-2	19.80	21.00	0.00	75.05	0.00	22.37	8.98	1.38	0.47	16.55	14.30
BJ-2	14.46	45.70	0.00	30.56	0.00	18.01	5.39	1.30	0.25	15.86	13.57
HF-1	16.38	23.05	0.00	71.90	0.00	23.50	5.85	1.86	1.24	13.80	2.52
HF-1	6.00	6.31	0.80	34.88	0.00	24.75	22.60	2.61	0.32	10.70	12.38
YX-1	16.54	31.31	0.00	78.70	0.00	29.53	19.01	5.15	1.05	10.45	6.01
YX-1	5.99	14.67	0.00	0.15	0.00	12.95	2.33	0.37	0.47	8.63	6.92
GZ-1	9.70	8.04	0.00	14.51	0.00	17.99	2.66	1.59	0.72	6.57	14.10
GZ-1	8.30	2.04	0.00	4.51	0.00	14.72	1.25	0.91	0.53	2.53	11.51
GZ-2	5.77	12.30	0.00	42.90	0.00	19.45	1.84	1.95	0.82	7.24	8.40
GZ-2	4.06	11.86	0.00	36.67	0.00	14.82	1.57	2.22	0.47	4.52	7.59

Supplementary Table S10 The concentration of ions and TOC in aerosols at sampling points of 3.0WS (TOC: total organic carbon; 3.0WS: Sampling points located 3.0m above the water surface).

3.0WS	Cl^{-} (μg/m^3)	NO_3^{-} (μg/m^3)	NO_2^{-} (μg/m^3)	SO_4^{2-} (μg/m^3)	PO_4^{3-} (μg/m^3)	Na^{+} (μg/m^3)	NH_4^{+} (μg/m^3)	K^{+} (μg/m^3)	Mg^{2+} (μg/m^3)	Ca^{2+} (μg/m^3)	TOC (μg/m^3)
BJ-1	21.80	0.00	2.70	51.90	0.00	0.00	0.00	0.00	0.00	0.00	1.74
BJ-1	0.00	0.00	3.81	0.07	0.00	0.00	0.00	0.00	0.00	0.00	6.45
BJ-1	0.00	25.91	2.50	67.37	0.00	18.43	0.45	0.00	0.00	12.35	15.00
BJ-2	9.09	26.25	0.00	74.69	3.57	0.00	0.00	0.00	0.00	0.00	18.20
BJ-2	11.20	11.90	2.44	34.90	0.00	0.00	0.00	0.00	0.00	0.00	13.70
BJ-2	4.23	0.00	0.00	0.00	0.00	22.77	12.83	2.33	0.00	18.78	15.30
HF-1	6.76	21.10	2.51	97.30	0.00	25.60	5.49	2.00	1.40	14.30	2.28
HF-1	5.85	4.06	0.00	0.00	0.00	1.50	5.34	0.57	0.00	3.85	14.92
YX-1	6.93	28.00	0.00	59.08	0.00	23.95	19.37	3.67	1.01	9.08	5.59
YX-1	2.15	4.80	0.00	0.00	0.00	2.16	2.24	0.30	0.00	2.34	14.39
GZ-1	6.21	13.50	0.00	21.10	0.00	19.10	2.04	1.64	0.75	6.69	16.88
GZ-1	5.02	11.30	0.00	21.34	0.00	0.93	3.56	1.12	0.00	3.03	12.96
GZ-2	6.57	19.00	0.00	17.57	3.60	14.70	3.48	2.50	1.02	8.85	13.28
GZ-2	5.24	17.00	0.00	17.60	3.60	3.87	4.19	1.27	0.31	7.59	11.20

Supplementary Table S11 The concentration of ions and TOC in aerosols at sampling points of 1.5WS (TOC: total organic carbon; 1.5WS: Sampling points located 1.5m above the water surface).

1.5WS	Cl^- (μg/m^3)	NO_3^- (μg/m^3)	NO_2^- (μg/m^3)	SO_4^{2-} (μg/m^3)	PO_4^{3-} (μg/m^3)	Na^+ (μg/m^3)	NH_4^+ (μg/m^3)	K^+ (μg/m^3)	Mg^{2+} (μg/m^3)	Ca^{2+} (μg/m^3)	TOC (μg/m^3)
BJ-1	20.81	11.20	0.00	20.40	13.84	5.89	2.82	1.53	2.39	2.10	2.23
BJ-1	15.80	2.05	0.00	2.56	1.22	9.21	3.79	2.67	1.67	3.59	3.44
BJ-1	11.42	16.70	0.00	24.01	1.30	11.28	1.57	0.89	6.21	2.50	15.07
BJ-2	10.50	24.40	0.00	71.50	6.13	22.17	5.15	1.74	0.32	12.84	42.47
BJ-2	10.24	42.80	0.00	66.30	0.70	25.75	7.24	3.75	0.10	8.70	10.96
BJ-2	20.10	3.15	0.00	2.10	0.80	16.14	3.29	0.99	0.05	9.40	15.50
HF-1	5.58	17.43	0.00	77.62	3.30	20.42	4.05	1.67	0.95	10.14	1.89
HF-1	30.66	4.83	0.00	6.46	1.70	1.85	6.23	0.93	0.14	0.27	16.17
YX-1	5.57	40.3	0.00	69.75	1.30	25.13	17.02	4.10	0.08	8.71	5.31
YX-1	2.61	4.52	0.00	1.50	0.34	1.75	2.12	0.22	0.10	2.79	10.30
GZ-1	4.96	3.70	0.00	20.20	3.34	13.00	0.10	0.76	0.70	6.14	7.49
GZ-1	24.45	1.20	0.00	0.20	0.58	24.54	6.46	27.83	0.50	5.27	12.60
GZ-2	4.56	1.70	2.40	0.90	1.50	24.00	1.10	1.25	0.85	8.29	6.71
GZ-2	2.30	7.58	0.00	10.92	1.80	20.92	1.60	15.22	1.33	11.89	15.79

Supplementary Table S12 The concentration of ions and TOC in wastewater (TOC: total organic carbon).

Wastewater	Cl^- (μg/L)	NO_3^- (μg/ L)	NO_2^- (μg/ L)	SO_4^{2-} (μg/ L)	PO_4^{3-} (μg/ L)	Na^+ (μg/ L)	NH_4^+ (μg/ L)	K^+ (μg/ L)	Mg^{2+} (μg/ L)	Ca^{2+} (μg/ L)	TOC (μg/ L)
BJ-1	121.00	54.10	0.00	152.00	35.00	86.38	1.80	22.80	5.10	62.10	156.00
BJ-1	80.00	1.60	6.35	94.00	15.00	79.27	1.32	21.70	2.40	54.75	54.20
BJ-1	92.70	0.80	7.20	101.00	8.70	96.14	0.60	25.72	4.70	26.70	25.68
BJ-2	137.76	53.71	0.00	153.00	18.00	95.85	0.90	57.10	5.90	67.62	113.00
BJ-2	124.05	55.10	0.00	126.70	3.50	89.14	2.37	21.49	7.50	59.20	87.60
BJ-2	73.30	6.06	0.00	0.00	1.60	76.63	1.20	27.00	4.30	61.80	33.70
HF-1	156.04	40.20	0.00	128.00	1.50	84.66	0.80	12.62	9.44	59.10	109.00
HF-1	191.97	54.02	2.41	0.00	2.80	96.91	0.59	20.99	4.99	50.78	25.68
YX-1	172.00	35.00	2.89	121.00	1.90	121.52	10.56	63.65	23.00	82.36	115.00
YX-1	194.61	37.10	0.00	57.80	2.10	123.60	0.85	5.40	3.86	60.37	34.50
GZ-1	75.00	8.70	0.00	64.00	3.40	96.00	1.60	11.20	6.65	58.30	160.00
GZ-1	68.96	7.99	0.00	2.07	1.50	65.87	0.80	13.19	8.90	43.66	23.28
GZ-2	88.00	10.60	0.00	74.34	2.00	26.20	1.30	17.80	7.95	42.90	67.00
GZ-2	57.22	49.26	0.00	38.00	1.20	28.24	0.90	24.17	6.22	40.72	27.85
